# 肺腺癌IASLC/ATS/ERS国际多学科分类以及临床实践中应注意的问题

**DOI:** 10.3779/j.issn.1009-3419.2013.07.01

**Published:** 2013-07-20

**Authors:** 利娟 闫, 祥华 易

**Affiliations:** 1 471003 洛阳，洛阳市第二中医院病理科 Department of Pathology, Luoyang Second Hospital of Traditional Chinese Medicine, Luoyang 471003, China; 2 200065 上海，同济大学附属同济医院，上海市同济医院病理科 Department of Pathology, Tongji Hospital, Tongji University School of Medicine, Shanghai 200065, China

**Keywords:** 肺肿瘤, 分类, 病理学, 肿瘤学, Lung neoplasms, Classification, Pathology, Oncology

## Abstract

2011年由国际肺癌研究协会（International Association for the Study of Lung Cancer, IASLC）、美国胸科学会（American Thoracic Society, ATS）和欧洲呼吸学会（European Respiratory Society, ERS）发表的肺腺癌国际多学科分类共识意见，首次提出了分别适用于手术切除标本、小活检及细胞学的分类方法，不再使用细支气管肺泡癌的名称，新增原位腺癌和微浸润性腺癌的命名，对浸润性腺癌提倡全面而详细的组织学诊断模式等。随着临床应用的深入，病理和临床医师对新分类的理解和应用在某些方面尚存在一定的困惑。本文对这一肺腺癌的国际多学科分类新标准进行解读并就临床实践中所应该注意的问题进行分析和讨论。

## Abstract (en)

The international multidisciplinary classification of lung adenocarcinoma sponsored by International Association for the Study of Lung Cancer/American Thoracic Society/European Respiratory Society in 2011, addresses the classification which is appropriate for resection specimens, small biopsies and cytology, respectively for the first time. For resection specimens, new concepts are introduced such as adenocarcinoma *in situ* (AIS) and minimally invasive adenocarcinoma (MIA); Invasive adenocarcinomas are recommended to be classified by comprehensive histologic pattern. During the clinical practice, pathologists and clinicians have some confusions in understanding and application of to the new guideline to some extent. This paper will interpret this new guideline, discuss and analyze the issues that need to be noticed in clinical application.

2011年国际肺癌研究协会（International Association for the Study of Lung Cancer, IASLC）、美国胸科学会（American Thoracic Society, ATS）和欧洲呼吸学会（European Respiratory Society, ERS）联合在《胸部肿瘤学杂志》（*J Thorac Oncol*）上发表了关于肺腺癌国际多学科分类（以下简称新分类）^[1]^。新分类一经提出就引起了我国临床和病理医师的极大关注，通过一年多的推广和使用，基本得到了临床和病理医师的理解和接受。随着应用的日益广泛，病理和临床医师对新分类的理解和应用在某些方面尚存在一定的困惑。本文主要对这一新分类标准进行解读，并就临床实践中所应该注意的问题进行分析和讨论。

## 1 肺腺癌新分类提出的背景

肺癌是世界上发病率和死亡率最高的恶性肿瘤之一，而肺腺癌是肺癌最常见的组织学类型，约占所有肺癌的一半左右。肺腺癌不同的组织亚型在临床、影像学、病理学和遗传学上有很大的差异。近年来，尽管对这一类肿瘤的基础和临床研究取得了明显进步，但仍然需要对肺腺癌亚型有一个国际上普遍接受的标准。原来的肺癌分类，包括1967年、1981年和1999年WHO分类主要是由病理学家参与制定的病理分类，缺乏多学科的融汇和贯通，如非小细胞肺癌（non-small cell lung cancer, NSCLC）的病理学分类对患者的治疗和预后没有重大的指导作用。2004年WHO分类尽管增加了遗传学和临床的信息^[2]^，但这些分类已不能很好地反映肿瘤分子生物学、病理学和影像学的新进展，也不能满足临床治疗和预测预后的需要。近10年肺腺癌分子生物学和影像学等取得了明显的进步，新的诊断技术和治疗药物以及新的治疗模式不断诞生，迫切需要一个肺癌的多学科分类以满足临床诊治和研究的需求。为此，IASLC、ATS和ERS于2011年发表了肺腺癌的国际多学科分类。新分类由多个国际著名学会联合，有内科（肿瘤）医师、放射医师、病理医师、胸外科医师和分子生物学专家参与。新分类首次提出了分别适用于手术切除标本、小活检及细胞学的分类方法; 不再使用细支气管肺泡癌（bronchioloalveolar carcinoma, BAC）的名称，新增原位腺癌（adenocarcinoma *in situ*, AIS）和微浸润性腺癌（minimally invasive adenocarcinoma, MIA）的命名; 对浸润性腺癌提倡全面而详细的组织学诊断模式等，目标是制定一个对患者治疗及预后更有意义的肺腺癌病理分型。适用于手术标本的肺腺癌新分类的类型：

### 1.1 浸润前病变

#### 1.1.1

非典型腺瘤性增生;

#### 1.1.2

原位腺癌（原来≤3 cm的BAC）：①非粘液性; ②粘液性; ③粘液和非粘液混合型。

### 1.2

微浸润性腺癌（≤3 cm以伏壁样生长方式为主且浸润灶≤5 mm的小腺癌）：①非粘液性; ②粘液性; ③粘液和非粘液混合型。

### 1.3 浸润性腺癌

#### 1.3.1 伏壁状为主（原来的非粘液性BAC，浸润灶 > 5 mm）

#### 1.3.2 腺泡状为主

#### 1.3.3 乳头状为主

#### 1.3.4 微乳头状为主

#### 1.3.5 实性为主伴有粘液产物

### 1.4 浸润性腺癌的变异型

#### 1.4.1 浸润性粘液腺癌（原来的粘液性BAC）

#### 1.4.2 胶样癌

#### 1.4.3 胎儿型腺癌（低度恶性和高度恶性）

#### 1.4.4 肠型腺癌

## 2 肺腺癌新分类的特点

与2004年WHO肺癌的组织学分类相比^[2]^，肺腺癌的新分类有以下特点：

### 2.1 首次提出了分别适用于手术切除标本、小活检及细胞学的分类方法

手术切除标本的肺腺癌分为四种基本类型：浸润前病变（preinvasive lesions, PL）、MIA、浸润性腺癌和浸润性腺癌的变异型。

### 2.2 推荐不再使用BAC的名称，首次提出AIS和MIA的命名

将非典型腺瘤样增生（atypical adenomatous hyperplasia, AAH）和AIS一起归入肺腺癌的浸润前病变。

#### 2.2.1 废除BAC这一诊断术语

2004年WHO分类对BAC的诊断作了严格的规定，只有肿瘤细胞沿肺泡结构生长，即伏壁状生长（lepidic growth）（也有译为贴壁状生长），而无间质、血管或胸膜浸润证据才能诊断为BAC。然而在病理诊断中所用的“BAC”包括了孤立性小的非浸润性周围型肿瘤、微小浸润性腺癌、沿肺泡壁生长为主的浸润性腺癌、混合性亚型浸润性腺癌和广泛播散性粘液腺癌。这些从非浸润性肿瘤、低度恶性肿瘤到高度恶性肿瘤都归为“BAC”，给临床诊治和研究造成很大的混乱，也给癌症流行病学研究带来困难。因此，新分类废除了“BAC”这一诊断术语。

#### 2.2.2 首次提出AIS的概念，与AAH一起归入肺腺癌的PL

AAH的诊断标准与2004年WHO分类相同。其病变局限、病灶≤0.5 cm，增生的细胞为肺泡Ⅱ型细胞和/或Clara细胞，衬覆于肺泡壁或者呼吸性细支气管管壁上，细胞轻至中等异型。影像学上，AAH通常为≤0.5 cm的磨玻璃样结节（ground glass nodule, GGN）。其预后好，可长期稳定不变，临床上一般不需要处理，通常每年CT随访一次。

AIS相当于原来≤3 cm的BAC，其被定义为≤3 cm的局限性小腺癌，肿瘤细胞完全沿肺泡壁生长，无间质、血管或胸膜浸润。肺泡间隔可增宽伴硬化，但无瘤细胞间质浸润，肺泡腔内无瘤细胞聚集，无瘤细胞形成乳头或微乳头生长方式。AIS可分为非粘液性、粘液性和粘液/非粘液混合性三种。大部分AIS为非粘液性，由肺泡Ⅱ型上皮和/或Clara细胞组成，粘液性AIS罕见。AIS完全切除后预后极好，5年无瘤生存率达100%。在影像学上，AIS的典型表现一般为GGN，在HRCT上此AAH的密度稍高，有时病变表现为部分实性结节，偶为实性结节。粘液性AIS常表现为实性结节或实变。

#### 2.2.3 提出MIA的概念

MIA被定义为孤立性、以伏壁样生长方式为主且浸润灶≤0.5 cm的小腺癌（病灶≤3 cm）。病变内可以是1个或多个≤0.5 cm浸润灶（多个浸润灶以最大直径浸润灶为准，不是将多个大小不等浸润灶的直径相加）。大多数MIA为非粘液型，粘液型罕见。浸润成分判断的标准是：①肿瘤细胞除沿肺泡壁生长外，还有腺癌的其它组织学亚型（即腺泡、乳头、微乳头和/或实性）成分; ②肿瘤细胞浸润到肌纤维母细胞性间质中。注意，如果肿瘤内出现淋巴管、血管或胸膜侵犯以及出现肿瘤性坏死时，不能诊断为MIA，应直接诊断为浸润性腺癌。影像学上，MIA表现不一，非粘液性MIA通常表现为以磨玻璃样成分为主的部分实性结节，实性成分位于病变中央，≤0.5 cm。粘液性MIA很少见，表现为实性或部分实性结节。影像学定义CT上的结节其最大直径≤3 cm，如病变 > 3 cm则称为肿块。影像学上结节≤3 cm的阈值与病理诊断AIS或MIA最大直径一致。MIA完全切除后预后好，5年无瘤生存率接近100%。新分类中确定AIS和MIA的大小为≤3 cm，但实际上大多数AIS和MIA < 2 cm，更大的结节多数是浸润性腺癌。

### 2.3 浸润性腺癌分类的变化

浸润性腺癌分为以伏壁状（贴壁状）、腺泡状、乳头状、实性生长方式为主的亚型，推荐新增“微乳头状”亚型，将原WHO分类中透明细胞腺癌、印戒细胞腺癌归入实性为主亚型。

#### 2.3.1 新分类不再推荐使用混合性亚型浸润性腺癌

70%-90%手术切除的肺腺癌为浸润性腺癌，其中约80%由多种组织学亚型混合组成，新分类推荐按腺癌中最主要的组织学亚型分类，而不再使用混合性亚型。原来的非粘液性BAC主要以沿肺泡壁生长方式，如肿瘤浸润灶最大直径 > 0.5 cm，则诊断为伏壁状为主（lepidic predominant）的浸润性腺癌，其它亚型分别为腺泡状为主、乳头状为主、微乳头为主和实性为主伴有粘液产物的浸润性腺癌。浸润性腺癌按主要的组织学亚型命名，如果肿瘤内其它亚型成分 > 5%（而不是以前所采用的 > 10%），也应在病理报告中注明，并报告各亚型所占百分比。

#### 2.3.2 伏壁状为主的腺癌（lepidic predominant adenocarcinoma, LPA）

由肺泡Ⅱ型细胞和/或Clara细胞组成，肿瘤细胞沿肺泡壁表面生长，形态学相似于AIS和MIA，但浸润灶至少一个最大直径 > 0.5 cm时诊断为LPA。浸润的定义同MIA，除了伏壁状生长方式外，还有腺泡状、乳头状、微乳头状和/或实性生长方式，肿瘤细胞浸润到肌纤维母细胞性间质。前面已经提及，如有淋巴管、血管和胸膜侵犯以及肿瘤性坏死，应诊断为LPA，而不是MIA。需要注意的是，LPA只能用于以伏壁状生长为主的非粘液性腺癌，而不是以伏壁状生长为主的浸润性粘液腺癌，这与MIA不同，后者偶可以是粘液性MIA。将LPA作为浸润性腺癌的一个独立亚型，还由于与其它浸润性腺癌相比，其预后较好，Ⅰ期LPA的5年无复发生存率达95%。

#### 2.3.3 微乳头为主浸润性腺癌

2004年WHO分类中没有将该型列为独立的亚型。最近研究显示以微乳头成分为主的腺癌具有较强的侵袭行为，易发生早期转移，与实性为主腺癌一样，预后很差。微乳头为主腺癌的肿瘤细胞小，立方形，以缺乏纤维血管轴心的乳头状方式生长，这些微乳头可附着于肺泡壁上或脱落到肺泡腔内，常有血管和间质侵犯。

#### 2.3.4 透明细胞腺癌和印戒细胞腺癌

2004年WHO分类将这两种腺癌列为浸润性腺癌的特殊变型，实际上具有透明细胞和印戒细胞特点的腺癌见于多种组织学亚型的浸润性腺癌。新分类认为这些细胞学改变不足以构成一种特殊的组织学亚型，而归入浸润性腺癌的实性为主亚型，如果在其它组织亚型的腺癌中出现透明细胞或印戒细胞，应予以报告其成分和百分比。

### 2.4 浸润性腺癌其它变型的变化

浸润性腺癌的变异型包括浸润性粘液型腺癌（原来的粘液型BAC）、胶样型腺癌、胎儿型腺癌、肠型腺癌。

#### 2.4.1 取消“粘液性囊腺癌”，将其归入胶样腺癌

2004年WHO分类中的“粘液性囊腺癌”非常少见，可能是胶样腺癌形态学谱系中的一员，新分类不再单独列出，而将其归入胶样腺癌。

#### 2.4.2

原来的透明细胞腺癌和印戒细胞腺癌归入实性为主亚型。

#### 2.4.3 新增加肠型腺癌（enteric adenocarcinoma）

这是新分类中增加的一种新的变型，肺的原发性肠型腺癌由具有结直肠腺癌某些形态学和免疫表型特点的成分所组成，且肠分化成分占肿瘤的50%以上。肠型腺癌可有其它肺腺癌组织学亚型成分如沿肺泡壁生长，免疫表型至少可表达一种结直肠癌的标记物如CDX2、CK20或MUC2。半数肠型腺癌病例可表达细胞角蛋白7（cell keratin 7, CK7）和甲状腺转录因子-1（thyroid transcription factor 1, TTF-1），可以与转移性结直肠癌相鉴别。

#### 2.4.4 胎儿型腺癌

由富含糖原、无纤毛细胞的小管（类似胎儿肺小管）组成的腺体所构成的特殊类型腺癌，细胞内常有核下空泡，腺腔内可见鳞状样桑椹体。分为低度恶性和高度恶性两类，大多数为低度恶性，预后较好。与高度恶性相比，低度恶性者Wnt信号转导通路中的成分如β-连环蛋白（β-catenin）上调。此型与其它亚型混合出现时，应按照优势成分进行分类。

## 3 肺腺癌新分类对临床和病理的一些建议和规定

### 3.1 新分类对小活检和细胞学诊断肺癌所做的一些规定

大约70%肺癌患者在做病理诊断时已属晚期或发生转移，只能通过小活检和细胞学标本做诊断，因此新分类标准提供了较详细的针对小活检和细胞学标本的指导。

① 肺癌组织学具有明显的异质性，小活检和细胞学标本不可能反映整个肿瘤的组织学结构（亚型），是否存在浸润也难以判断。因此，小活检和细胞学标本不能诊断“AIS”和“MIA”，同理，大细胞癌也无法依据小活检或细胞学作出诊断。此类诊断须建立在对肿瘤进行全面取材的基础上。

② 如果病理医生不能在光镜的基础上对肿瘤进行明确分类，则应该借助于免疫组化和/或组织化学染色等来进一步分类，同时在病理报告中要注明分类是在进行免疫组化或组织化学染色的基础上得出的诊断。应该尽可能地少用组织学类型不明确的非特指性NSCLC（NSCLC-not otherwise specified, NSCLC-NOS）这一术语。

③ 大多数NSCLC单独依据形态学能做出腺癌或鳞状细胞癌的诊断，对于小活检标本是一个分化差NSCLC，可以依据纯形态学提示临床倾向腺癌或鳞状细胞癌，需注明没有做特殊染色或免疫组织化学染色。约10%-30%的NSCLC分化差，小活检和/或细胞学标本难以进一步分型，通常诊断为NSCLC-NOS。要借助于免疫组织化学TTF-1和p63等尽可能将NSCLC区分为倾向腺癌和倾向鳞状细胞癌，以便选择靶向药物治疗。

④ 小活检和细胞学标本除用于病理诊断外，还应适当留存一些标本做基因突变、扩增和染色体易位等分子检测，用于预测某些靶向药物治疗的疗效。

⑤ 新分类推荐细胞学检查最好与小活检组织学检测一起进行，以提高诊断的准确性。

### 3.2 表皮生长因子受体（epidermal growth factor receptor, *EGFR*）基因突变的检测是非常必要的

对晚期肺腺癌应该进行*EGFR*基因突变的检测，因为有*EGFR*基因突变的晚期肺腺癌患者用酪氨酸激酶抑制剂（tyrosine kinase inhibitors, TKIs）厄洛替尼（erlotinib）或吉非替尼（gefitinib）治疗均有明显疗效，无进展生存与对照组相比明显延长。

### 3.3 尽可能使用明确的诊断名称

由于近年在肺癌化疗上的进展，应尽可能地使用腺癌或鳞状细胞癌这样明确的诊断名称; 不管是临床还是病理，都要尽可能地少用NSCLC这个诊断术语。要借助于免疫组化和组织化学等技术尽可能将NSCLC区分为倾向腺癌和倾向鳞状细胞癌，以便选择靶向药物治疗。因为晚期肺腺癌对多靶点抗叶酸药物培美曲赛（pemetrexed）和抗血管内皮生长药物贝伐珠单抗（bevacizumab）治疗仍然有效，而鳞状细胞癌对培美曲赛治疗效果不如腺癌，用贝伐珠单抗治疗可引起鳞状细胞癌患者致命性的大出血。

### 3.4 分子标记物的检测有利于肺腺癌的评估和治疗

这些标记物的检测除了*EGFR*基因突变外，还有*KRAS*基因突变、*EGFR*基因扩增和棘皮动物微管相关蛋白样4-间变淋巴瘤激酶（echinoderm microtubule associated protein like 4-anaplastic lymphoma kinase, *EML4-ALK*）基因融合的检测，对药物反应的预测有一定价值。最近有资料显示印戒细胞数量 > 10%的实性为主腺癌患者中高达56%肿瘤有*EML4*和*ALK*基因的融合*EML4-ALK*，有些易位的肿瘤与*EGFR*和*KRAS*基因突变相互排斥，ALK抑制剂克立唑替尼（crizotinib）对有ELM4-ALK易位的肺腺癌治疗有效。

### 3.5 分子标记物的检测应有计划性

要充分地利用活检组织进行病理学诊断和分子分析，对活检组织病理标本要妥善处理和保存。尤其是对小活检和细胞学标本，应该提供尽可能多的、高质量的组织来进行分子研究。

## 4 肺腺癌新分类中有待解决的问题

肺腺癌新分类还存在一些不足之处，与病理诊断和预后相关的有待解决的问题主要涉及下面几个方面：

① 由于冰冻切片取材的局限性等因素，病理医师尚不能通过冰冻切片区分MIA和AIS，此时临床医师应该理解。

② MIA的诊断标准是基于有限的数据，需进一步的循证医学的资料确认。包括测量浸润成分范围的最佳方法，浸润灶以0.5 cm为阈值是不是最佳的选择？如果出现多处浸润，应使用最大浸润灶的最大径线，还是病灶总大小乘以浸润成分的百分比？疤痕大小或主要的间质粘连及炎症如何影响浸润成分范围？与非粘液性肿瘤相比，粘液性MIA的诊断标准是否不同？

③ 符合MIA诊断标准的肿瘤，若浸润成分以实体性、微小乳头状为主，或若出现巨细胞及梭形细胞成分但又不符合多形性癌的诊断，也有100%无疾病生存率吗？

④ 微小乳头状类型腺癌在晚期和早期病例中是否都意味着较差的预后？若侵袭性微小乳头状或实体性成分不代表主要的类型而以相对少量的形式出现，是否存在预后意义？或它们占多少比例才有意义？

⑤ 应用突变型*EGFR*特异性抗体的免疫组化检测是否能够预测*EGFR*基因的突变尚不明确。

⑥ 肺腺癌新分类仍不能推荐任何明确的分级系统，需更多的研究以确定最佳的分级系统是否应包括组织结构、细胞核评估或两者的结合。

此外，由于肺癌的异质性，腺癌往往与鳞状细胞癌、神经内分泌癌以及肉瘤样癌等混合存在，此时的肺癌分类和诊断标准有待细化和完善。我们知道，任何一个肿瘤组织学分类都不可能解决该肿瘤诊断的全部问题，肺腺癌新分类难免会有些不足。正是因为有如此多的问题需要解决，所以才有更多研究与思考的空间，使攻克肺癌这个顽固的堡垒更富挑战性。

## 5 肺腺癌新分类诊断中的体会和应注意的问题

### 5.1 病理医师要充分熟悉肺腺癌新分类的诊断标准，以便准确把握和应用新分类进行肺癌的诊断

例如，新分类建议不要使用BAC这一诊断名称，在诊断“AIS”时最好附上2004年WHO分类中“原来的BAC”; 在诊断“伏壁状为主腺癌”时附上“混合性亚型腺癌，由BAC和腺泡成分混合”。利用小活检和细胞学标本做诊断时，应尽可能按临床要求提供免疫组织化学和分子检测信息。肺腺癌新分类中涉及原来的BAC概念应该注意区别。

① AIS：主要为原来非粘液性的BAC，极少为粘液性。

② MIA：主要为原来非粘液性的BAC，极少为粘液性。

③ LPA：原来非粘液性的BAC。

④ 腺癌：侵袭为主伴有一些非粘液性伏壁样成分（包括一些之前分为混合亚型的切除肿瘤及一些之前归为非粘液性BAC的临床晚期腺癌）。

⑤ 浸润性粘液型腺癌：原来粘液性BAC。

### 5.2 新分类对浸润性腺癌提倡全面、详细的组织学诊断模式，而不再笼统地将其归为混合亚型

诊断模式举例：肺腺癌，以乳头性为主，10%呈腺泡样生长，5%呈实体性生长。新分类推荐，如果肿瘤内其它亚型成分 > 5%即应在病理报告中描述，而不是以前WHO所推荐的 > 10%的标准。

### 5.3 病理医师要了解新分类相关的临床和影像学知识，与临床医师和影像科医师互相沟通，扬长避短，以提高肺癌的病理诊断水平

例如：在冷冻切片上，病理医师有时很难准确区分AAH、AIS和MIA，而不同的诊断直接关系到外科医师的手术方式和范围。影像学上纯磨玻璃影有助于AAH或AIS的诊断，而部分实性结节则有助于MIA的诊断。

### 5.4 尽管新分类对各种类型肿瘤的诊断标准做了明确规定，但在实际工作中还会遇到一些问题，需要通过临床实践不断完善

例如，MIA的浸润灶最大直径≤0.5 cm，取材时要通过最大切面进行直径的测量。如果肿瘤组织周围继发出现肺泡萎陷、炎症实变和间质纤维化时可能影响最大直径的观察。新分类中有些标准还缺乏足够的循证医学支持。例如前面已经提及，MIA的单个浸润灶最大直径≤0.5 cm作为与浸润性腺癌区分的阈值是否恰当等。

### 5.5

在缺乏免疫组化、组织化学和分子检测的情况下，肺腺癌的诊断和分型只能完全依靠病理形态学来评价NSCLC显示神经内分泌的形态学特点而无法做神经内分泌免疫组化学标记时，可诊断为大细胞癌，并描述具有神经内分泌形态特点，可能为大细胞神经内分泌癌，需要做免疫组化进一步证实。要仔细进行HE切片的光镜观察，对于实性腺癌，应仔细寻找胞浆内的粘液，如果能在个别细胞胞浆内找到粘液，也可以做出明确诊断。

### 5.6 新分类的优势

新分类较好地体现了病理形态学、免疫表型、分子生物学与临床和影像学的结合，不仅有利于肺腺癌各亚型的病理诊断和鉴别诊断，而且对治疗和预后的判断有较大的帮助。例如，浸润性粘液性腺癌与非粘液性AIS、MIA和LPA在临床和免疫表型等方面不同：后者女性较多见，影像学主要表现为磨玻璃影，细胞类型为Ⅱ型肺泡上皮和/或Clara细胞，CK7阳性（98%）和半数以上TTF-1阳性（67%），大约半数有*EGFR*突变，*KRAS*突变较低（大约为13%）。

总之，肺腺癌新分类是国际多学科合作的产物，与过去单纯的病理组织学分类不同，它是结合病理形态学、免疫组织化学和分子生物学，并融入临床和影像学资料的结晶，能更好地为临床诊断和分类提供依据。新分类通过细化临床诊断、深化肿瘤分子标志物研究和指导晚期肺腺癌的内科治疗，无疑将使肺腺癌的诊断、治疗和科研再上一个新台阶。病理医师在应用该分类时应积极地从多学科、多角度综合分析，最终做出更加全面和准确的病理诊断。在使用这个新分类时，病理医师和临床医师以及影像科医师要相互沟通、相互学习，取长补短，在实践中发现问题，不断提高，为新分类的完善和提高积累更多循证医学的资料。
